# Whole-genome sequencing reveals genomic diversity and selection signatures for adaptation in South African Afrikaner and Bonsmara cattle

**DOI:** 10.3389/fgene.2026.1717538

**Published:** 2026-02-17

**Authors:** D. Alberts, E. van Marle-Köster, F. Joubert, D. P. Berry

**Affiliations:** 1 Department of Animal Science, University of Pretoria, Pretoria, South Africa; 2 Centre for Bioinformatics and Computational Biology, Department of Biochemistry, Genetics and Microbiology, University of Pretoria, Pretoria, South Africa; 3 Animal & Grassland Research and Innovation Centre, Teagasc, Moorepark, Ireland

**Keywords:** candidate genes, gene ontology, genetic structure analysis, indigenous cattle, short-read sequences

## Abstract

The indigenous Afrikaner and composite Bonsmara cattle breeds are hardy and adapted to the diverse South African climate and biomes. Both breeds have been successfully used in the South African stud and commercial industries. This study explored the genomic diversity and population structure, as well as identified selection signatures within and between the Afrikaner and Bonsmara breeds with a focus on signatures related to adaptation traits. Short-read whole genome sequencing data of 42 Afrikaner and 43 Bonsmara cattle were analysed. Diversity analysis revealed comparable nucleotide diversity levels in the Afrikaner and Bonsmara populations, with the Bonsmara having weaker average linkage disequilibrium between adjacent single nucleotide polymorphisms as well as having fewer runs of homozygosity. Furthermore, genetic structure analysis revealed distinct clustering of both populations, with the exception of a subset of Afrikaner individuals having been infused with Bonsmara genetics. Between and within breed selection signatures were detected using the fixation index and integrated haplotype score approaches, respectively. Several gene ontology terms were described based on the detected selection signatures, with the most significant being nervous system development and multicellular organismal processes. Finally, functional annotation of the candidate genes from the within-breed selection signature analysis revealed several genes (*B3GLCT*, *HSPA2*, *HSPH1*, *STING1*) relating to adaptive traits in both populations. The location of the within and between breed selection signatures in this study population is consistent with the performance and adaptive characteristics of both breeds and may enhance future breeding strategies with the inclusion of these breeds in crossbreeding programs. Furthermore, a comprehensive genomic characterization of these breeds through whole genome sequencing data is important as these adapted breeds are valuable reservoirs of genetic variation.

## 1 Introduction

South Africa hosts several genetically diverse cattle breeds farmed under varying climatic conditions characterized by high average summer temperatures, erratic rainfall, and seasonal variability in veld quality and quantity (Van Marle-Köster, 2013; Deacon and Black, 2021). Natural and artificial selection over centuries has resulted in indigenous breeds and locally developed composites that have acquired adaptive characteristics for these production conditions (Visser et al., 2021). A major challenge for the Southern African region is to produce sufficient animal protein, while at the same time considering the future impact of climate change and global warming (Ramírez-Ayala et al., 2021). Therefore, local breeds are of particular interest given their adaptive and productive traits as a means of contributing to delivering on the growing demand for animal protein (FAO, 2012; Biscarini et al., 2015).

African cattle can be classified into different types, namely, the *Bos taurus taurus* (African taurine), *Bos taurus indicus* (also known as zebu), sanga (hybrids of *Bos taurus taurus* and *Bos taurus indicus*), and zenga (typically sanga and zebu backcrosses) (Mwai et al., 2015). Afrikaner cattle are classified as Sanga and are regarded as one of the oldest indigenous breeds in South Africa, which originated from the Koi people during the 17th and 18th centuries (Scholtz et al., 2016). It is a medium framed animal and is yellowish to red in color and is known to be resistant to various diseases, parasites and temperatures (Makina et al., 2014; Scholtz et al., 2016). Furthermore, Afrikaner cattle have been used as a founder breed for several composite breeds in South Africa such as the Bonsmara, Hugenoot, South African Braford, and Sanganer. There has, however, been a sharp decline in the number of registered Afrikaner animals since the 1980s due to feedlots favouring the faster growth rate of *Bos taurus* breeds (Steenkamp and Tissier, 2016; Pienaar et al., 2018). To improve the production potential and retain the favourable traits of the Afrikaner, genetic infusion of the Bonsmara breed was pursued. The infusion project was initiated in 1991, with Bonsmara semen being used in an Afrikaner herd (Vermaak et al., 2016).

The Bonsmara composite breed was developed between 1937 and 1963 in the pursuit of a South African adapted beef breed, while simultaneously demonstrating good fertility, growth, and carcass qualities, which are typical characteristics of the *Bos taurus* beef breeds (Bonsma, 1980; Bonsma, 1985; Bonsmara, 2022). At the time of development of the South African Bonsmara, the Santa Gertrudis breed had been developed in America with a composition of five-eighths Shorthorn and three-eighths Brahman (Santa Gertrudis Breeders’ Society of South Africa, 2017). It became apparent that when the taurine breed percentage exceeded 50%, the cattle showed signs of distress, increased respiration rates, and ultimately hyperthermia in tropical climates (Bonsma, 1985). The primary challenge faced when British and European beef breeds were brought to South Africa, was tropical degeneration (i.e., the effect of climatic stress), which led to very high mortality rates (Bonsma, 1985). The program for the Bonsmara was initiated to include local Sanga (i.e., Afrikaner) and British breeds, which resulted in a final composition of five-eighths Afrikaner and three-eighths British beef breeds, namely, the Hereford and Shorthorn (Bonsma, 1980). The Bonsmara breed society operates an open herd book, where founder animals are continuously added using a structured upgrading program; this strategy is contributing to a larger population size (Bonsmara, 2022). Numerically, the Bonsmara is considered one of the largest beef breeds in South Africia with approximately 91,000 registered cows (FAO DAD-IS, 2025). The breed supports compulsory participation in animal recording for fertility, growth, and efficiency traits (Van Marle-Köster and Visser, 2018). Both the Afrikaner and Bonsmara breeds are known to have a lower susceptibility to heat stress and tick-borne diseases compared to the European and British breeds farmed in South Africa (Foster et al., 2009; Bonsmara, 2022).

Advances in genomics over the past 2 decades have made routine single nucleotide polymorphism (SNP) genotyping possible as well as the generation of whole genome sequences; the latter, in particular, provides opportunities for deeper insights into within and across-breed genomic variation and selection signatures, among others (Fleming et al., 2018). Whole genome sequencing technologies provide a means of bypassing the ascertainment bias associated with SNP genotyping panels. Furthermore, with whole genome sequencing, all genome-wide SNPs (the number being reliant on sequencing depth/coverage) are captured and is not limited to the variants on the genotyping panels. Sequence data, therefore, enables higher-resolution insights into the existing genome-level diversity of indigenous breeds and may be especially beneficial for exploring rare genetic variants (Eusebi et al., 2020). In addition, sequence data increases the power and precision in locating the causative mutations underlying monogenic defects, rare variants and genomic regions subject to selection pressures (Georges et al., 2019; Eusebi et al., 2020). Consequently, whole-genome sequencing enables more precise characterisation of genomic composition and facilitates the identification of breed-specific SNPs across breeds and species.

Both natural and artificial selection contribute to changes in genetic variation at specific loci and at linked neutral loci (de Simoni Gouveia et al., 2014; Kooverjee et al., 2022). The resulting genomic footprints following selection, known as selection signatures, provide information on domestication and evolutionary processes, as well as functional information of genes and genomic regions (de Simoni Gouveia et al., 2014). Additionally, signatures of selection enable the identification of candidate genes underlying traits of economic importance and are of key interest to animal geneticists (Saravanan et al., 2020). Signatures of selection can be detected using different methods, such as site frequency spectrum, linkage disequilibrium (LD) patterns, reduced local genomic variability, single-site differentiation, or haplotype-based differentiation (Saravanan et al., 2020). The fixation index (F_ST_) reflects the differences in allele frequencies between populations and has been used in numerous cattle studies to identify genomic regions under selection (Gibbs et al., 2009; Qanbari et al., 2010; Saravanan et al., 2020). The integrated haplotype score (iHS) test is an extension of the extended haplotype homozygosity method (Sabeti et al., 2007; Saravanan et al., 2020) and is based on LD (Voight et al., 2006). Both these methods are used to detect selection signatures - the F_ST_ for interpopulation exploration and the iHS for intrapopulation exploration.

Genomic studies of local South African breeds have been limited to using SNP genotyping panels, thus potentially suffering from SNP ascertainment bias (Makina et al., 2016; Reding et al., 2023). The Afrikaner and Bonsmara represent unique genetic resources, presenting an opportunity to genomically characterize the diversity of these breeds and explore selection signatures important for both selection and adaptation (Friedrich et al., 2024). The Afrikaner and Bonsmara breeds are well recognized for their adaptation to South Africa’s diverse climatic conditions (Scholtz et al., 2016; Bonsmara, 2022). This adaptability underscores the importance of investigating traits related to adaptation, such as thermoregulation and immune response in these breeds. We hypothesized that these breeds would show distinct selection signatures in genomic regions associated with thermoregulation, immune response, and heat tolerance - traits critical for adaptation to South African climates. Through the utilization of low coverage whole genome sequencing data, a more accurate characterization of the genomic architecture underpinning adaptive traits is possible, mitigating the ascertainment bias typically associated with SNP array panels. In the present study, low coverage whole genome sequencing data were used to conduct genomic characterization of the South African Afrikaner and Bonsmara populations as well as detect possible selection signatures related to adaptation.

## 2 Materials and methods

### 2.1 Data origin

Samples for DNA extraction were obtained using RFID tags for sampling ear tissue. Afrikaner and Bonsmara cattle born after 2009 were included, selected from stud breeders in the Grassland and Savanna biomes, which represent the typical South African climate (Mucina and Rutherford, 2006). The most influential, unrelated animals with genetic merit indices above average were selected from 16 stud breeders in total for the Afrikaner and Bonsmara breeds, located across South Africa. Although larger sample sizes and higher coverage sequencing data are generally recommended for comprehensive genome studies, the scope of this study was constrained by available financial resources; a decision was made to include more animals with lower coverage that fewer animals with higher coverage. The sample size is comparable with the Zwane et al. (2021) study, in which 30 Afrikaner, 30 Drakensberger, and 30 Nguni animals were included, illustrating that smaller cohorts are sometimes unavoidable due to financial constraints, especially in developing countries such as South Africa. The Afrikaner and Bonsmara breed societies granted permission to generate the whole genome sequence data, and ethical approval was granted by the University of Pretoria (UP) (NAS264/2021) for the analyses of external data. The ear tissue samples were extracted with QIAGEN DNA blood and tissue kits at the University of Pretoria and sent to Ludwig-Maximilian University, Munich, for sequencing. Sequencing data of 42 Afrikaner (AFR) and 43 Bonsmara (BON) animals were generated at low coverage (approximately 1X to 3X) and received in VCF format, following the GATK best practices pipeline, from Prof Laurent Frantz (Ludwig von Maximillian University, Munich).

### 2.2 Mapping and sorting

Quality control and trimming were performed on the raw reads in FastQC v0.11.7. The Burrows-Wheeler Alignment tool (BWA) (Li and Durbin, 2009) was used to create an index and, thereafter, BWA mem was used to map the clean pair-end reads to the *Bos taurus* reference genome (ARS-UCD1.2) using default parameters (Rosen et al., 2020; Picard toolkit, 2019). Metrics were calculated on the aligned sequences with Picard’s “CollectAlignmentSummaryMetrics” tool. Furthermore, the Picard version 2.17.11 “*MarkDuplicate*” command-line utility (Picard Tools- By Broad Institute, https://broadinstitue.github.10/picard/) was used to remove sequence duplicates for the BAM files. Validation of BAM files was undertaken using the Picard “ValidateSamFile” tool.

### 2.3 Variant calling, annotation, and VCF file preparation

The “*BaseRecalibrator*” and “*PrintReads*” command-line tools of the Genome Analysis Toolkit v4.2.6.1 (GATK) (van der Auwera et al., 2013) were used for Base Quality Score Recalibration (BQSR) before variant calling (https://gatk.broadinstitute.org/hc/en- us/articles/360037055712-ApplyBQSR). The “*HaplotypeCaller*” command-line utility of the GATK v4.2.6.1 toolkit was used for concurrently calling SNPs and small insertions and deletions (InDels) through *de novo* haplotype assembly. Subsequently, the GATK “*GenotypeGVCFs*” command line was used for joint genotyping of the pre-called samples. Thereafter, the GATK “*SelectVariants*” mode was used to separate InDels and SNPs into two different files before hard filtering (“*VariantFiltration*”) each variant type to reduce false positive variants. Finally, SnpEff (Cingolani et al., 2012a) and SnpSift (Cingolani et al., 2012b) were used for variant annotation and functional effect prediction.

All InDels were removed with the--remove-indels command of VCFtools v0.1.16 (Danecek et al., 2011), and the file was bgzipped and tabix indexed (Li, 2011). Thereafter, the file was subjected to the “-m2 -M2 -v snps” options of the BCFtools file manipulation software to only retain bi-allelic SNPs (Danecek et al., 2021). As the variants of the animals were jointly called, there was only one output VCF file, with combined information on all the breeds.

### 2.4 Quality control and genomic diversity

VCFtools version 0.1.16 (Danecek et al., 2011) was used to estimate nucleotide diversity (π) per breed, with a window size and step size of 50 kb in both instances; nucleotide diversity was plotted with the boxplot function in R (R Core Team, 2023). LD decay may reveal population recombination history, through the recombination rate and number of generations of recombination (Zhang et al., 2019). LD decay for both breeds was estimated and visualized with the multi-population commands in the PopLDdecay software (Zhang et al., 2019). The merged dataset as well as the individual Afrikaner and Bonsmara datasets were converted into PLINK file format through PLINK v.2.0 (Chang et al., 2015). After retaining only bi-allelic and autosomal SNPs, more than 119 million SNPs remained. Following LD pruning, 7,980,947 SNPs for the full dataset of both breeds (merged), 5,928,777 SNPs for the Afrikaner dataset, and 7,249,230 SNPs for the Bonsmara dataset remained. The--indep-pairwise method with thresholds 50 5 0.8 was applied, with the first parameter being a window size (in kilobases), the second being the variant count to shift the window, and the last parameter representing the upper *r* ^2^ threshold.

Both MAF and heterozygosity statistics were estimated to reflect the extent of genetic variability in both breeds. High heterozygosity values within a population could be indicative of low sample quality, whereas low heterozygosity values could be indicative of inbreeding (Marees et al., 2018). The minor allele frequency (MAF) for the autosomal SNPs was calculated in PLINK v2.0 with the--freq command and the proportion of SNPs in different frequency categories per breed were plotted as histograms with the ggplot function in R (Wickham, 2016). The average observed (H_O_) and expected heterozygosity (H_E_), and method-of-moments inbreeding coefficients (F_IS_) were calculated for each breed separately with the--het function in PLINK v.2.0. The F_IS_ statistic was calculated as: (1-(observed het. count/expected het. count)).

### 2.5 Population structure and phylogeny

Genetic relatedness and population structure between the Afrikaner and Bonsmara populations were inferred using principal component analysis (PCA) and admixture performed on the full dataset using the SNPs common to both breeds following LD pruning. A genomic relationship matrix was constructed, and the principal components were estimated with the--make-grm-list and--pca commands, respectively, using PLINK v2.0 (Chang et al., 2015). PCA is frequently applied in population genetics studies to explore patterns in genomic variability by geographic regions and ancestral background, capturing the most genetic variation within the first few principal components (McVean, 2009). In the present study, the first ten principal components were used in the PCA, to collectively capture the most significant variance and meaningful patterns. The PCA plot was visualized using a scatterplot. Ancestral population components were determined using ADMIXTURE, considering either K = 2 to K = 4, with a lowest cross validation error observed at K = 2 (0.373) (Alexander and Lange, 2011). The admixture plots were visualized with the Genesis v. 0.2.6 software (Buchmann and Hazelhurst, 2014).

The matrix of Hamming distance between pair-wise individuals was calculated for phylogenetic reconstruction using the--distance function in PLINK v.1.09 (Purcell et al., 2007). Furthermore, this function provides a distance matrix in which the total mismatched allele count across all SNPs for a pair of individuals is provided. For two individuals (i and j), Dij was calculated as:

$$Dij=\sum_{k=1}^{M}diff\left(M_{ik},M_{jk}\right)$$

where M is the total number of non-missing SNPs; M_ik_ and M_jk_ is the genotype of individual i and j at marker k; and diff(M_ik_,M_jk_) is the absolute difference in allele counts between the two individuals at marker k. Subsequently, the matrices were converted into text (.txt) files and used in R (R Core Team, 2023) for the construction of a neighbor joining tree using the ‘ape’ package (Saitou and Nei, 1987; Paradis and Schliep, 2019). Thereafter, it was converted into Newick format and uploaded onto iTOL (Letunic and Bork, 2024) for graphical representation of the neighbor joining tree. Runs of homozygosity (ROHs) were identified using the--homozyg command in PLINK v.1.09 using--homozyg-snp 90, and--homozyg-snp 98 for the Afrikaner and Bonsmara populations, respectively (Purcell et al., 2007). The--homozyg-snp was calculated with:

$$l=\frac{\log_{e\;}\frac{a}{n_{s}*n_{i}}}{\log_{e}\left(1-het\right)}$$

where l is the minimum number of SNPs required in a window, a is the false positive rate, $n_{s}$ the number of SNPs in the dataset, $n_{i}$ the number of individuals, and het the average heterozygosity across all SNPs. The number and length (in Mb) of ROH for each individual per population were estimated and divided into four categories: 0–2 Mb (ancient inbreeding), >2–4 Mb (historical inbreeding), >4–8 Mb (more recent inbreeding), and >8 Mb (recent inbreeding).

### 2.6 Identification of selection signatures

Although low coverage may reduce power to detect rare variants and affect phasing accuracy, stringent filtering and complementary analyses (i.e., F_ST_ and iHS) were employed to maximize reliable signal detection. Selection signatures were identified between the Afrikaner and Bonsmara breeds using the F_ST_ unbiased estimator approach per SNP (Weir and Cockerham, 1984). By comparing allele frequencies, the F_ST_ statistic was used to detect between-breed genetic differences suggestive of strong, intentional selection pressures. The top 0.1% most significant windows were considered as candidate selection signatures, and only SNPs with an absolute F_ST_ values greater than 0.5 were retained. For the detection of intrapopulation selection signatures, the Integrated Haplotype Score (iHS) test statistic was implemented with the ‘rehh’ package in R (Gautier et al., 2017). This statistic explores haplotype structure within breeds to identify recent positive selection. Phasing of all 85 animals was undertaken with the Beagle v5.5 software package (Browning et al., 2021) using default parameters (i.e., number of phasing iterations = 5, sliding window length defined by genetic distance = 5,000) suitable for large datasets. For the detection of the candidate regions, the top 0.1% most significant windows were considered, where the iHS value had to be > 3. The iHS test focuses on regions in which beneficial alleles are at an intermediate frequency but are not yet fixed, thereby detecting incomplete selection signatures (Voight et al., 2006).

### 2.7 Gene annotation and functional analysis

SNPs detected using the F_ST_ method to be under selection or candidate regions identified using iHS were required to reside within or overlap with genes to be considered for further annotation. For both the F_ST_ and iHS analyses, the genes were annotated with the GALLO package in R (Fonseca et al., 2020; R Core Team, 2023). To gain insight into the gene functions and signaling pathways of the candidate genes identified, the online Kyoto Encyclopedia of Genes and Genomes (KEGG) pathway and Gene Ontology (GO) were performed using DAVID 2021 and ShinyGo v.0.82 (Huang et al., 2009; Ge et al., 2020; Sherman et al., 2022). Thereafter, genes involved in immune response and adaptation-related functions were identified and compared with currently available literature; genes common to both selection detection methods were also focused on. The highest and lowest iHS values were identified as novel signatures of selection and subsequently mapped to the ARS-UCD1.2 cattle reference genome to determine their proximity to annotated genes. Additionally, the genes proximal to the novel selection signatures detected in the present study were compared with the scientific literature to determine whether they had any immune and adaptation-related roles in cattle.

## 3 Results

### 3.1 Sequencing data

Based on the sequencing metrics, a total yield of 11.55 billion mapped reads was achieved. A mean mapping quality score of 14.08, a mean GC content of 46.21%, and average insert size of 120.16 base pairs were observed. High local alignment was observed, with a representative subsample showing 33.85 million reads (99.75% of million total reads) and an associated million duplicated reads. The mean coverage of the sequencing data, however, was 1.72X suggesting a limitation in sequencing depth, and a shallow distribution of reads across target regions.

### 3.2 Genomic diversity

**FIGURE 1:**
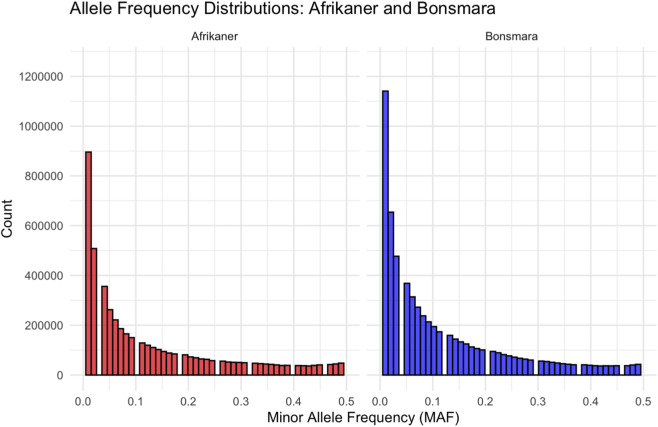
Minor allele frequency distribution for the Afrikaner and Bonsmara populations at sequence level.

**FIGURE 2:**
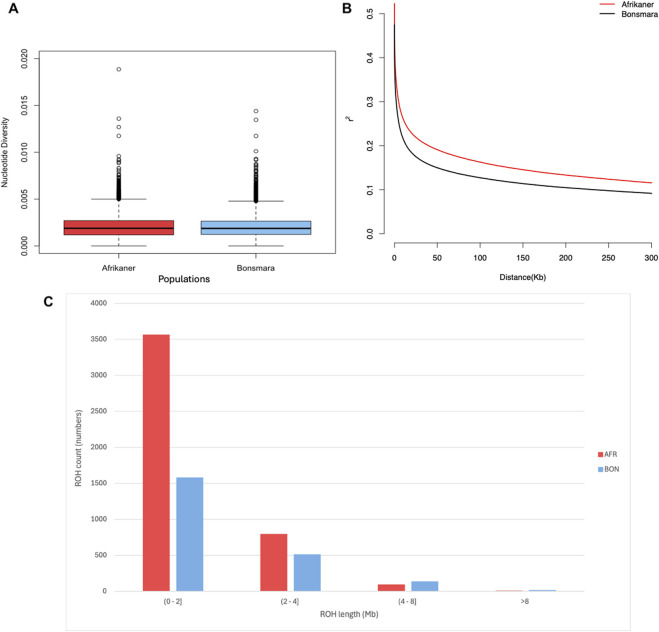
Patterns of genomic variation in the Afrikaner (AFR) and Bonsmara (BON). **(A)** Genome-wide nucleotide diversity (π) with a 50 kb step size. **(B)** Genome-wide average linkage disequilibrium decay for each breed. **(C)** Runs of homozygosity distribution of various lengths in the Afrikaner and Bonsmara.

In both cattle populations, a high prevalence of rare alleles was observed as represented on the allele frequency distribution plot (Figure 1). The Bonsmara has a much higher initial peak in the low MAF range and a higher curve across most of the low to mid frequency range, indicative of larger number of markers and larger effective population size. The observed heterozygosity was higher than the expected heterozygosity in both cattle breeds, with low average inbreeding coefficients, indicating an excess in diversity (Table 1). The Afrikaner had a slightly higher average observed heterozygosity (*H*_*O*_ = 0.282) compared to the Bonsmara (*H*_*O*_ = 0.260), while both had low average inbreeding coefficients. Within the two populations, the nucleotide diversity (π) was comparable and consistent with the similarity in both mean heterozygosity and mean inbreeding levels per breed (Figure 2A). The Bonsmara had a weaker average linkage disequilibrium between adjacent SNPs compared to the Afrikaner (Figure 2B). The majority of ROH detected in both cattle populations were <2 Mb in length, indicating ancient inbreeding, while both breeds of cattle displayed similar levels of recent inbreeding (Figure 2C).

**TABLE 1:** The average heterozygosity and the average, lowest and highest inbreeding coefficients per breed n - sample number; HO - observed heterozygosity; HE - expected heterozygosity; FIS - inbreeding coefficient average.

Breed	n	Average H_O_	Average H_E_	Average F_IS_	Lowest F_IS_	Highest F_IS_
Afrikaner	42	0.282	0.218	−0.011	−0.180	0.070
Bonsmara	43	0.260	0.204	−0.011	−0.080	0.040

### 3.3 Population structure and phylogeny

**FIGURE 3:**
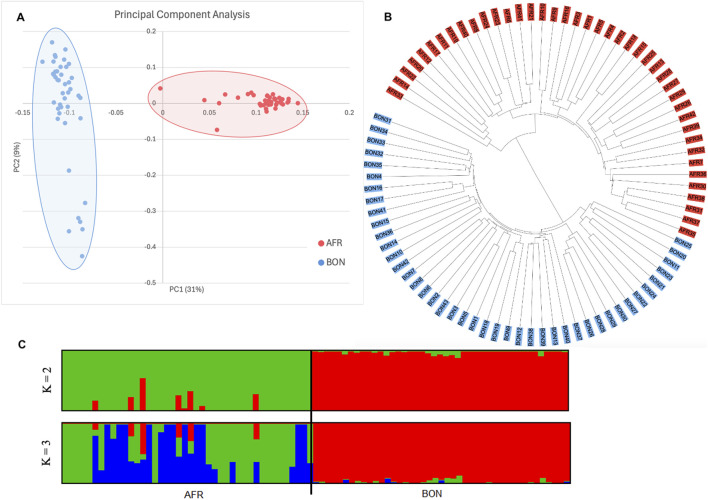
Population structure of the two breeds. **(A)** Principal component analysis (PC1 vs. PC2) of the Afrikaner and Bonsmara. **(B)** Neighbor-joining tree of the AFR and BON with Nei’s pairwise genetic distances. **(C)** Admixture bar plots of the two populations with K = 2 and K = 3, lowest cross-validation error at K = 2.

The principal component analysis indicated two separate clusters (Figure 3A) which was also confirmed by the circular neighbor-joining tree separating the Afrikaner population from the Bonsmara population (Figure 3B). The Afrikaner population split into two distinct clades within the neighbor-joining phylogenetic tree indicating that five individuals were genetically divergent from the majority of the Afrikaner cattle included in this analysis. The Bonsmara population was also stratified into two different clades.

For the ADMIXTURE analysis, K = 2 had the lowest cross-validation error (0.373). The Bonsmara has developed into a distinct breed (Figure 3C), yet shares some ancestry with the Afrikaner. The highest level of admixture was detected in the Afrikaner population with the potentially infused Afrikaner animals observable in Figure 3C.

### 3.4 Detection of selection signatures

**FIGURE 4:**
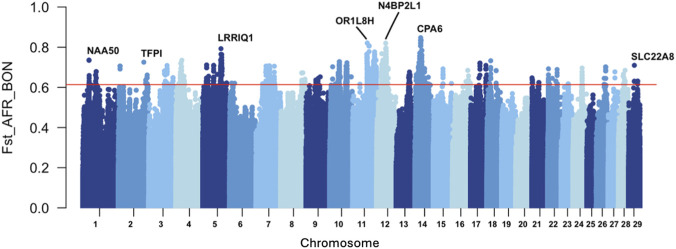
Manhattan plot of the top F_ST_ values for the between population comparison of the Afrikaner (AFR) and Bonsmara (BON), with the top 0.1% considered as candidate genes.

The Manhattan plot of the absolute F_ST_ values comparing the Afrikaner and Bonsmara breeds is presented in Figure 4. Of the candidate SNPs identified in the between-population analysis, peaks were observed on chromosomes 5, 11, 12, and 14. In these peaks, the SNPs exhibiting the highest F_ST_ values were located within the genes *LRRIQ1*, *OR1L8H*, *N4BP2L1*, and *CPA6*, respectively.

KEGG pathway enrichment analysis of the genes identified through the F_ST_-based SNP differentiation between Afrikaner and Bonsmara cattle revealed significant involvement of pathways including the MAPK signaling pathway, cellular senescence, oocyte meiosis, autophagy, parathyroid hormone synthesis, secretion, and action, T cell receptor signalling pathway, dopaminergic synapse, viral carcinogenesis, and the Hippo signalling pathway (Huang et al., 2009; Sherman et al., 2022). Collectively, these pathways contribute towards fundamental biological processes such as immune response, cell fate, reproduction and disease management (Zhang and Liu, 2002; Glick et al., 2010; Jaffe and Egbert, 2017). Upon investigation of the candidate regions obtained from the F_ST_ analysis, several candidate genes were identified and found to be associated with immune-related functions and traits (Supplementary Table S1).

**FIGURE 5:**
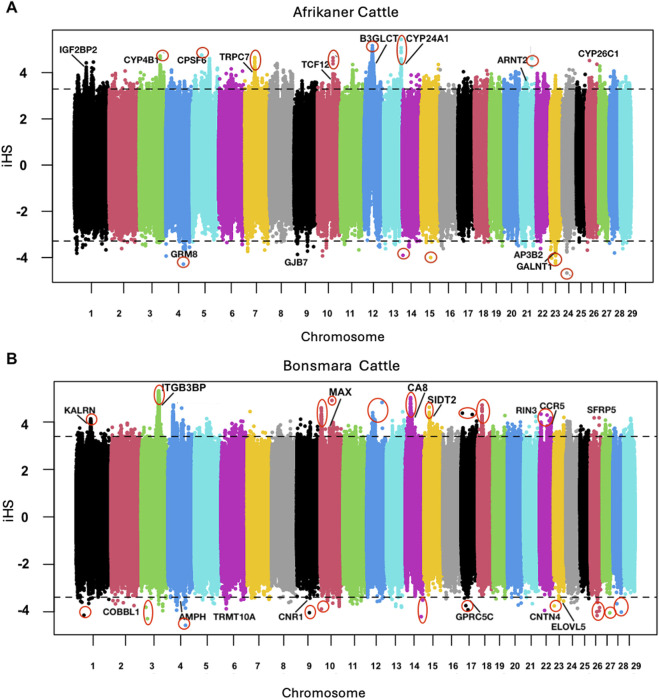
Manhattan plots of the top iHS values for the within-population statistical test. **(A)** Afrikaner (AFR) population. **(B)** Bonsmara (BON) population.

The Manhattan plots of the Afrikaner iHS and Bonsmara iHS estimates are in Figures 5A,B, respectively. The highest peaks for the Afrikaner were on chromosomes 5, 7, 12 and 13, overlapping the genes *CPSF6*, *TRPC7*, *B3GLCT*, and *CYP24A1*. The lowest iHS values in the Afrikaner were on chromosomes 4, 23, and 24. Moreover, the highest iHS in the Bonsmara were on chromosomes 3, 10, 14, and 15, within the genes *ITGB3BP*, *MAX*, *CA8*, and *SIDT2* and the lowest values were on chromosomes 3, 4, 14, and 26. Several of these putative selection signatures have not previously been reported in cattle (circled in red).

For the iHS analysis in both the Afrikaner and Bonsmara breeds, GO terms involved genes related to immunity and adaptation (Table 2). In the Afrikaner, the most significant terms were associated with nervous system development (GO:0007399), tissue development (GO:0009888), and anatomical structure morphogenesis (GO:0009653) (Supplementary Figure S1). Furthermore, in the Bonsmara population, the most significant terms were associated with hyperosmotic response (GO:0006972), positive regulation of glial cell proliferation (GO:0060252), and cellular response to estradiol stimulus (GO:0071392) (Supplementary Figure S2). Several genes were identified with both detection methods, of which 12 were related to immunity and adaptation (Table 3).

**TABLE 2:** Candidate genes identified with integrated haplotype score associated with adaptation traits in the two cattle populations.

Role/Function	Gene name	Population	BTA	Position	References
Adaptation	*RGS3* *TRPC7*	AFR	8 7	102,829,115 47,655,137	Liu et al. (2021) Weldenegodguad et al. (2019)
​	*AQP1*	AFR, BON	4	65,433,648	Liu et al. (2021)
Base coat color	*HSPA2*	AFR, BON	10	76,648,760	Kunene et al. (2022)
Heat stress response	*HSPH1*	AFR, BON	12	29,796,159	Garner et al. (2020)
​	*ASIC3, HSPA14, HSPB3*	BON	4 13 20	113,625,093 29,492,741 24,638,190	Ben-Jemaa et al. (2020) Pavani et al. (2016) Sengar et al., 2018
Heat tolerance	*CMPK1* CPSF6* *GHR* *HSPB6* *OLA1*	AFR	3 5 20 18 2	98,887,962 44,560,279 31,869,704 46,466,906 22,528,224	Bang et al. (2024), Kumar et al. (2022)
​	*IL6* *INTS6* *NPFFR2* *OAS2 MAPK8IP1*	BON	4 12 6 17 15	31,454,757 20,816,690 87,248,962 61,337,090 75,918,875	Liu et al. (2020)
​	*DNAJC14 GRXCR1*	AFR, BON	5 6	57,439,666 61,696,746	Bahbahani et al. (2015), Saravanan et al. (2021)
Rectal temperature	*DGCR8* *GOT1* *KBTBD2* *LSM5* *TXNRD2*	BON	17 26 4 4 17	72,976,003 20,416,120 63,915,891 64,014,974 72,872,642	Dikmen et al. (2013), Otto et al. (2019)
Immune response	*FKBP1A*	AFR, BON	13	59,737,061	Saravanan et al. (2021)
Thermal stress; preslaughter stress	*HSPBAP1*	BON	1	67,051,022	Cassar-Malek et al. (2022), Sengar et al. (2018)
Mandibulofacial dysostosis	*CYP26C1*	AFR	26	14,403,229	Sieck et al. (2020)

^a^ Gene positions measured in base pairs (bp); AFR, Afrikaner; BON, Bonsmara; BTA, *Bos taurus* autosome.

**TABLE 3:** Candidate genes relating to adaptation overlapping between the fixation index and integrated haplotype score analysis in the Afrikaner and Bonsmara populations.

Population	BTA	Position^a^	Gene name	Role	References
AFR	5	44,365,812	*LYZ2*	Host resistance to intestinal worm	Li et al. (2015)
​	11	104,590,862	*VAV2*	Tick resistance	Goyache et al. (2022)
​	12	29,625,632	*B3GLCT*	High-altitude adaptation and horn development	Flori et al. (2018), Terefe et al. (2022)
​	13	59,737,061	FKBP1A*	High-altitude pulmonary hypertension	Newman et al. (2011)
​	22	43,026,804	*ABHD6*	Heat tolerance	Luo et al. (2022)
​	28	29,285,721	*DNAJC9*	Cold adaptation	Weldenegodguad et al. (2019)
BON	1	29,106,840	*GBE1*	High altitude adaptation	Ayalew et al. (2024)
​	3	91,536,057	*TTC4*	BoHV-1 infection	Thompson et al. (2022)
​	10	7,6,612,009	*ZBTB1*	Immune cell regulation	Peixoto et al. (2023)
​	13	57,465,320	*GNAS*	Water reabsorption	Kim et al. (2020)
​	14	9,754,017	*ADCY8*	Eyelid pigmentation	Jara et al. (2022)
​	22	2,984,947	*RBMS3*	Spontaneous abortion	Suarez et al. (2025)

^a^ Gene positions measured in base pairs (bp); *Gene also present in the other population; AFR, Afrikaner; BON, Bonsmara; BTA, *Bos taurus* autosome.

## 4 Discussion

The sub-tropical regions of South Africa are characterized by high average summer temperatures, erratic rainfall, and seasonal variations in veld quality and quantity, making it important that breeds are selected suited to the specific climate (Deacon and Black, 2021; The World Bank Group, 2021). Sustainable management of these cattle populations is thus essential to ensure their continuous contribution to the livestock industry and breeds such as the Afrikaner and Bonsmara are of interest to South Africa due to their adaptive traits and production under extensive systems (Visser et al., 2021). Studies have been performed using SNP-based panels to estimate genomic diversity and selection signatures in the Afrikaner and Bonsmara breeds (Makina et al., 2016; Kooverjee et al., 2022; Ramoroka et al., 2025). To our knowledge, this is the first whole-genome sequencing report on the genomic diversity and detected signatures of selection in the Afrikaner and Bonsmara breeds, with a focus on genomic regions associated with adaptation. The animals included in the present study were selected to represent different South African biomes and animals representing herds with long term participation in official animal recording in South Africa.

### 4.1 Genomic diversity and population structure

The majority of the genetic variation for both breeds indicated a predominance for rare variants, which is supported by the low MAF values. The Bonsmara had a higher allele count across most of the MAF values, suggesting a larger effective population size. A similar allele frequency distribution pattern among African indigenous cattle was reported by Tijjani et al. (2024) with the use of sequence and high-density genotype arrays, highlighting the potential importance of rare alleles in shaping complex traits and maintaining genetic diversity within these populations.

The genomic diversity in the present study was moderate for both breeds with heterozygosity levels of 0.28 (Afrikaner) and 0.26 (Bonsmara). These results are consistent with previously reported heterozygosity values of 0.24 and 0.29 for the Afrikaner and Bonsmara breeds, respectively, based on BovineSNP50 data (Makina et al., 2014). Despite, low coverage sequencing data available for the present study, similar heterozygosity values were observed in Ethiopian breeds with higher coverage; Terefe et al. (2023) reported heterozygosity values of 0.27, 0.26 and 0.21 for the Afar, Boran and Kenana breeds, respectively. The inbreeding coefficients for both populations in the present study was comparable to previously reported inbreeding coefficients of 0.004 and −0.017 for the Afrikaner and Bonsmara, respectively using SNP data (Makina et al., 2014).

The Bonsmara exhibited weaker average LD in long and short distances compared to the Afrikaner population which can be attributed to them being composite breeds as composites tend to have greater genetic diversity compared to the pure breeds; a similar finding was reported by van der Nest et al. (2021) for the South African Simbra (a cross between the Brahman and Simmental cattle), with the use of genotype data from SNP panels. In the present study, the genomic diversity observed with low coverage sequencing data corresponds to the large population size of the Bonsmara breed, which allows for upgrading with its open herd book strategy.

The PCA and ADMIXTURE analyses in the present study based on low coverage sequencing data confirmed distinct clusters for the Afrikaner and Bonsmara populations, in agreement with previous analyses using SNP data (Makina et al., 2016). Shared ancestry was, however, visible on the structure plot, which confirms the Afrikaner ancestry in the Bonsmara composite breed. The six Afrikaner animals showing close relations to the Bonsmara animals can be explained by the infusion program used by the Afrikaner breed. The infusion project was initiated in 1991, where Bonsmara semen was used in an Afrikaner herd, repeated over 9 years, with the progeny backcrossed to the Afrikaner (Vermaak et al., 2016). The program was established to improve the growth potential and retain the favourable traits of the Afrikaner. Seven of the Bonsmara animals in the present study, which grouped independently, originate from a well-known family line in the breed and are all horned animals. Upon further inspection using the generated genomic relationship matrix, the average relationship among the seven Bonsmara animals (0.213) was approximately ten times higher than that of the entire Bonsmara population (0.027), suggesting these animals share one or more recent common ancestors.

### 4.2 Confirmation of known selection signatures

Based on the results from both the iHS and F_ST_ methods, the myostatin (*MSTN)* gene is under selection in the Bonsmara cattle population represented in the present study. The presence of the multiple *MSTN* variants, including Nt748, Nt414, and Q204X, has been confirmed in the South African Bonsmara (Bonsmara, 2022; Madula et al., 2024). The *MSTN* gene plays a key role in muscle development regulation (Dominique and Gérard, 2006) which is a trait that the Bonsmara breed is striving to improve. The deactivation of this gene causes double muscling in cattle (McPherron and Lee, 1997). The fact that the *MSTN* gene was detected to be under selection in the Bonsmara population in the present study confirms that the methods used in the present study for identifying selection signatures is appropriate and that low coverage sequencing data were sufficient for detection. In contrast, the *MSTN* gene was not observed to be under selection in the Afrikaner cattle in the present study, which is consistent with the lack of previous documentation of selection for this gene in the breed. The *CAPN1* gene, however, was found to be under selection in the Afrikaner breed corroborating previous studies on the meat tenderness of this breed being comparable, if not superior, to exotic breeds (Strydom et al., 2000; Strydom et al., 2016).

#### 4.2.1 Common genes under selection in both breeds

As climate change is expected to result in an increase in average temperatures and more frequent heat waves, selecting and breeding livestock with adaptive traits can assist in mitigation of the adverse impact of extreme climate events on animals (Cheng et al., 2022). The Afrikaner breed is known for having thick skin and a short, glossy coat, aiding with heat radiation (Scholtz et al., 2016), while the Bonsmara was bred using the Afrikaner as a base breed, inheriting the adaptive characteristics of the breed (Bonsma, 1980). In the present study, low coverage sequencing data were sufficient to identify candidate regions exhibiting preferential selection for adaptation in the Afrikaner and Bonsmara cattle genomes using the iHS statistical method. The iHS measure is suitable to detect signatures when selected alleles at intermediate frequencies (Voight et al., 2006; Saravanan et al., 2021). Demographic factors have minimal influence on this measure, reducing the likelihood of the occurrence of false positives (Voight et al., 2006; Saravanan et al., 2020). It is important to note that detection of iHS is more sensitive to genotype misclassification, which is common in low coverage sequencing data, since it relies heavily on phasing and accurate haplotype resolution. Heterozygotes might be called as homozygotes in low coverage data, which can reduce the power of detecting true signatures of selection (Baraja-Fonseca et al., 2025). Nevertheless, with stringent filtering and quality control, the utility of low coverage sequencing can be enhanced (Baraja-Fonseca et al., 2025).

Genes detected under selection that were common to both breeds include *BPIFB1* and *FKBP1A* (immune response), *AQP1* (adaptation), *DNAJC14* and *GRXCR1* (heat tolerance), *HSD17B7* (respiration rate), and *HSPH1* (heat stress response). The B3GLCT, MSRB3, and FRS2 genes were also identified as being under selection in both the Afrikaner and Bonsmara breeds. The *HSPH1* gene*,* which is on chromosome 12, has previously been reported to be under selection in the Bonsmara and Nguni breeds, and differentially expressed in the Holstein breed (Garner et al., 2020; Kooverjee et al., 2022). Interestingly, the *HSPA2* gene*,* which was under selection in both breeds in the present study has been associated with base coat colour in South African Nguni cattle using high-density SNP genotypes (Kunene et al., 2022). The *B3GLCT* gene, detected on chromosome 12, has previously been associated with high altitude adaptation and horn development in both cattle and sheep (Flori et al., 2018; Terefe et al., 2022). Selection on the *B3GLCT* gene, however, has not previously been reported in South African indigenous breeds, representing a novel discovery in these breeds.

The *MSRB3* and *FRS2* genes were found to be under selection in both breeds and have been documented to be under selection in other species. The *MSRB3* gene has been associated with ossification and fat deposition in Chinese cattle breeds but has also been associated with ear size and hearing in dogs and pigs (Chen et al., 2018; Li et al., 2023), suggesting a possible role in the ear size of cattle. In addition, the *FRS2* gene has been associated with immune response to *Eimeira maxima* jejunum infestations in chickens and has not been reported to be under selection in cattle, suggesting a novel discovery and possible contribution toward immunity in cattle (Jebessa et al., 2024).

#### 4.2.2 Genes detected to be under directional selection from the F_ST_ analysis

The F_ST_ method is a single site differentiation based method and measures the differences in allele frequencies between populations, with highly differentiated allele frequencies suggesting selection (Saravanan et al., 2021). The F_ST_ method highlights genomic regions possibly influenced by directional selection, but factors that can mimic selection signatures include population bottlenecks or expansions as well as genetic drift making it difficult to attribute regions detected by the F_ST_ analysis directly to selection (Saravanan et al., 2021). Additionally, Wright’s F_ST_ model assumes an idealized population of infinite size, which could result in inflated F_ST_ estimates when applied to small sample sizes like those in the present study. Nevertheless, as the number of SNPs increase, such as that with whole genome sequence data, the efficiency of detecting genetic differentiation also increases (Willing et al., 2012; Saravanan et al., 2020). Although several immunity genes show differentiation in selection between the Afrikaner and Bonsmara populations based on the F_ST_ analysis, these results should be cautiously interpretated and cannot confirm selection has acted to favor or disfavor specific genes in one population. Nevertheless, Lou et al. (2021) demonstrated that F_ST_ analysis can be conducted on low coverage whole genome sequencing when using stringent filtering and specialized analytical tools.

The F_ST_ method revealed several adaptation genes under selection in either the Afrikaner or the Bonsmara (Supplementary Table S1). The *STING1* gene, on chromosome 7, has been associated with thermogenesis in *Bos taurus* breeds and plays an important role in resistance to vector-borne diseases as an inflammatory response in African humped cattle (Kim et al., 2020; Huang et al., 2023). *MATR3,* also on chromosome 7, has been linked to local pathogen adaptation in African indicine cattle (Kim et al., 2020). *PTPN6,* on chromosome 5, plays a role in resistance to vector-borne diseases through innate and adaptive immunity and possibly in trypanotolerance in West African cattle (Tijjani, 2019; Kim et al., 2020). The *IL21* gene found on chromosome 17 has been involved in host-liver fluke interactions, relating to the Th17 cell differentiation pathway, and was suppressed during *Fasciola hepatica* infections in German dairy cows (Walsh et al., 2009; May et al., 2025). Finally, *CBLC* (chromosome 1) has been involved in the incidence of hoof and leg disorders in Braunvieh cattle and has been linked to arthritis in rats (Kosińska-Selbi et al., 2020).

#### 4.2.3 Selection signatures only present in either the Afrikaner or Bonsmara populations

Several adaptation-related genes were found to be under selection in just one of the two breeds investigated (Table 2). In the Afrikaner breed, the *CMPK1, CPSF6, GHR, HSPB6,* and *OLA1* genes were detected to be under selection and have previously been associated with heat tolerance in smallholder dairy cattle from Vietnam and Indian Karan Fries cattle (Kumar et al., 2022; Bang et al., 2024). The *RGS3* and *TRPC7* genes were directly related to adaptation in the Holstein cattle (Weldenegodguad et al., 2019; Liu et al., 2021). Furthermore, the *CYP26C1* gene was detected to be under selection in the Afrikaner population and a recessive mutation in this gene has been described as the cause for mandibulofacial dysostosis in Hereford calves (Sieck et al., 2020). Mandibulofacial dysostosis is a congenital condition causing facial deformities such as a shortened, asymmetric lower mandible and bilateral skin tags near the lips (Sieck et al., 2020). It would be of future interest to investigate whether this condition has been found in Afrikaner calves in South Africa, considering the genetic introgression from the Bonsmara breed into the Afrikaner population, and given that the Hereford breed is one of the foundation breeds contributing to the genetic makeup of the Bonsmara. As a gene relating to meat quality, the *CAPN1* gene was found to be under selection in the Afrikaner breed in the present study, confirming selection for meat tenderness in the breed (Strydom et al., 2000).

The *ASIC3*, *HSPA14*, and *HSPB3* genes detected to be under selection in the Bonsmara population (but not the Afrikaner) have been associated with heat stress response in North-African, dairy, and Frieswal cattle, respectively (Pavani et al., 2016; Sengar et al., 2018; Ben-Jemaa et al., 2020). The *DGCR8*, *GOT1*, *KBTBD2*, *LSM5*, and *TXNRD2* genes were found to be associated with rectal temperature in both Gir x Holstein crosses and purebred Holstein cattle (Dikmen et al., 2013; Otto et al., 2019). Furthermore, *IL6*, *INTS6*, *NPFFR2*, *OAS2*, and *MAPK81P1* have been related to heat tolerance in Holstein cattle (Liu et al., 2020). The *HSPBAP1* gene on chromosome 1 has been associated with both thermal- and preslaughter-stress in composite dairy and Normande cows, respectively (Sengar et al., 2018; Cassar-Malek et al., 2022). *ADCY8*, a gene previously associated with eyelid pigmentation in Hereford cows, which happens to be one of the base breeds in the Bonsmara breed, was detected on chromosome 14 (Jara et al., 2022). As mentioned previously, detecting the *MSTN* gene to be under selection in the Bonsmara breed in the present study confirms the phenotypic muscular hypertrophy occasionally reported in this breed (Madula et al., 2024). While many of these genes have been identified as being under selection in *Bos Taurus* cattle, including Holsteins, there is a noticeable lack of similar studies focused on the South African indigenous cattle breeds.

#### 4.2.4 Genes identified using both the detection methods

The candidate genes identified as being under selection that were common to both the iHS and F_ST_ methods are listed in Table 3. In the present study, the *LYZ2* gene was found to be under selection in the Afrikaner breed, which has been reported as strongly upregulated in Angus cattle and plays a role in host resistance to the *Cooperia oncophora* intestinal worm (Li et al., 2015). Further investigations with higher coverage sequencing data may be useful to confirm the role of *LYZ2* in Afrikaner cattle. The *VAV2* gene has been identified as a candidate gene in tick resistance in West African taurine cattle and was detected in the present study in the Afrikaner cattle, suggesting a role in the inherent resistance to ticks and tick-borne diseases in the breed (Afrikaner Cattle Breeders’ Society of South Africa, 2021; Goyache et al., 2022). Pulmonary hypertension has been linked to right heart failure in cattle residing in high-altitude regions. Moreover, some cattle breeds have heritable susceptibility to high altitude pulmonary hypertension, and the *FKBP1* gene has been identified as a candidate for pulmonary hypertension in Angus cattle (Newman et al., 2011). The *FKBP1* gene was identified as being under selection in both breeds in the present study.

The *DNAJC9* gene, observed under selection in the Afrikaner in this study, has been linked to cold adaptation in the Yakutian breed (Weldenegodguad et al., 2019). Although the Afrikaner breed is recognized for its hardiness and adaptation to warm climates (Scholtz et al., 2016), the impact of cold environments on this breed remains largely unexplored and the connection between the *DNAJC9* gene and the breed’s adaptability to colder conditions may require further exploration. The *TTC4* gene was observed under selection in the Bonsmara, and it has been reported that *TTC4* transcript levels increase during BoHV-1 infection, which causes infectious bovine rhinotracheitis and genital disease in cattle. Furthermore, increasing the expression of external *TTC4* leads to a rise in the production of infectious BoHV-1 virions (Thompson et al., 2022). A number of studies conducted have applied more than one test statistic for the detection of selection signatures, as different detection methods have their own characteristics (Ma et al., 2015). The present study, therefore, used both the F_ST_ and iHS methods for selection signature detection in the Afrikaner and Bonsmara breeds. Despite a low coverage of the whole genome sequencing data available, the common genes that have been detected by both methods show strong evidence of selection and underscore the importance of these genes for further studies in the two cattle populations.

#### 4.2.5 Genomic regions warranting further investigation

Several of the top signatures of selection identified have not previously been identified to be under selection in cattle but were in proximity to genes that have been implicated in adaptive traits. The *CMPK1* gene, mapped near position 3:98,858,924 in the Afrikaner cattle population, has previously been linked with thermotolerance (Edea et al., 2018). Similarly, the *TRGC3* gene, located near 4:82,720,073 in both the Afrikaner and Bonsmara breeds, has been associated with immunity in Tibetan cattle (Xia et al., 2025). Notably, *FBXW7*, found near 17:5,306,457, has been associated with diabetes in murine models and is a known tumor suppressor in humans (Zhao et al., 2018; Wang et al., 2024). Additionally, GCNT4, located near 10:6,484,903, is upregulated in response to *Ostertagia ostertagi* infections in cattle, where it participates in the synthesis of mucin core structures (Rinaldi et al., 2011). The close genomic association of these genes with adaptive traits underscores the importance of further investigation to elucidate their precise functional roles.

The presence of the genes detected to be under selection with whole-genome sequence data in the present study suggests their involvement in traits relating to adaptation such thermoregulation and immune response in the South African Afrikaner and Bonsmara cattle breeds. Despite the use of low coverage whole genome sequences, the results are similar to studies where SNP data were applied. Low coverage sequences overcome ascertainment bias observed when using SNP panels and can be used to effectively identify novel variation in underrepresented populations (Martin et al., 2021). Furthermore, it has been used to guarantee more than 99% of true positives and true negatives for traceability and parentage testing (Casellas et al., 2021). Low coverage sequencing has also been suggested as an unbiased and similarly priced alternative to genotype arrays in population genetics and polygenic score analysis. Nevertheless, one should take caution when using low coverage sequencing, as low read counts can result in genotype misclassification, a loss of rare variant detection, mapping issues, sequencing errors as variants, and bias in complex regions (Li et al., 2011; Thermofisher, 2025). In the future, validation using higher coverage sequencing or the use of independent populations will be essential to confirm these associations and to further strengthen the robustness of our findings.

There is a lack of studies using sequencing data in South African indigenous breeds for comparison and therefore this study serves as a benchmark. The identified signatures of selection in the present study provide insights into evolutionary pressures acting on these populations, highlighting genetic loci that possibly contribute toward immunity and adaptation thereby warranting further investigation. Key genomic regions and candidate regions under selection in the South African Afrikaner and Bonsmara cattle breeds were identified with the use of the F_ST_ and iHS methods applied to low coverage whole genome sequencing data. Selection signatures relating to adaptation traits such as thermoregulation, heat stress response and immune response were detected, highlighting the genetic potential of the Afrikaner and Bonsmara breeds to withstand the harsh climatic conditions of Southern Africa. The use of low coverage whole genome sequencing data enabled a more in-depth genomic characterization and detection of more selection signatures in indigenous South African cattle breeds than what has previously been achieved with SNP panels and is thus a cost-effective alternative to SNP panels. The findings provide valuable insight into the evolutionary pressures shaping these breeds and point to genomic regions warranting further investigation to improve breeding for climate resilience and productivity.

## 5 Conclusion

The present study utilized low coverage whole genome sequencing data to generate insights into the genetic diversity, population structure, and signatures of selection in the South African Afrikaner and Bonsmara cattle breeds. Several signals of selection were identified confirming adaptation of these breeds to the South African climate and highlights genomic regions with potential relevance to traits important to sustainability and productivity in the local beef industry. Both breeds exhibit high heterozygosity and limited inbreeding. Due to the small sample size and low sequencing coverage, findings should, nonetheless, be interpreted with caution; that said, the results underscore the importance of the use of whole genome sequencing for more comprehensive genetic characterization and selection signature detection. As sequencing costs continue to decline, higher coverage genomic studies of the indigenous South African cattle populations will be critical for genetic improvement programs. Overall, this research serves as a benchmark for sequencing of the indigenous South African breeds.

## Data Availability

The data analyzed in this study is subject to the following licenses/restrictions: The data was generated for the African Genomics project of the University of Pretoria. Consent acquired from the respective breeder societies. Requests to access these datasets should be directed to d.alberts@up.ac.za.
