# Supplementary material for: Whole-genome sequencing reveals genomic diversity and selection signatures for adaptation in South African Afrikaner and Bonsmara cattle
Source: Front Genet. 2026 Feb 17;17:1717538. doi: 10.3389/fgene.2026.1717538 (PMC12952722; doi:10.3389/fgene.2026.1717538)
Supplement: Supplementary file 1 [file DataSheet1.docx]

Supplementary Material

# Supplementary Figures and Tables

## Supplementary Figures

**
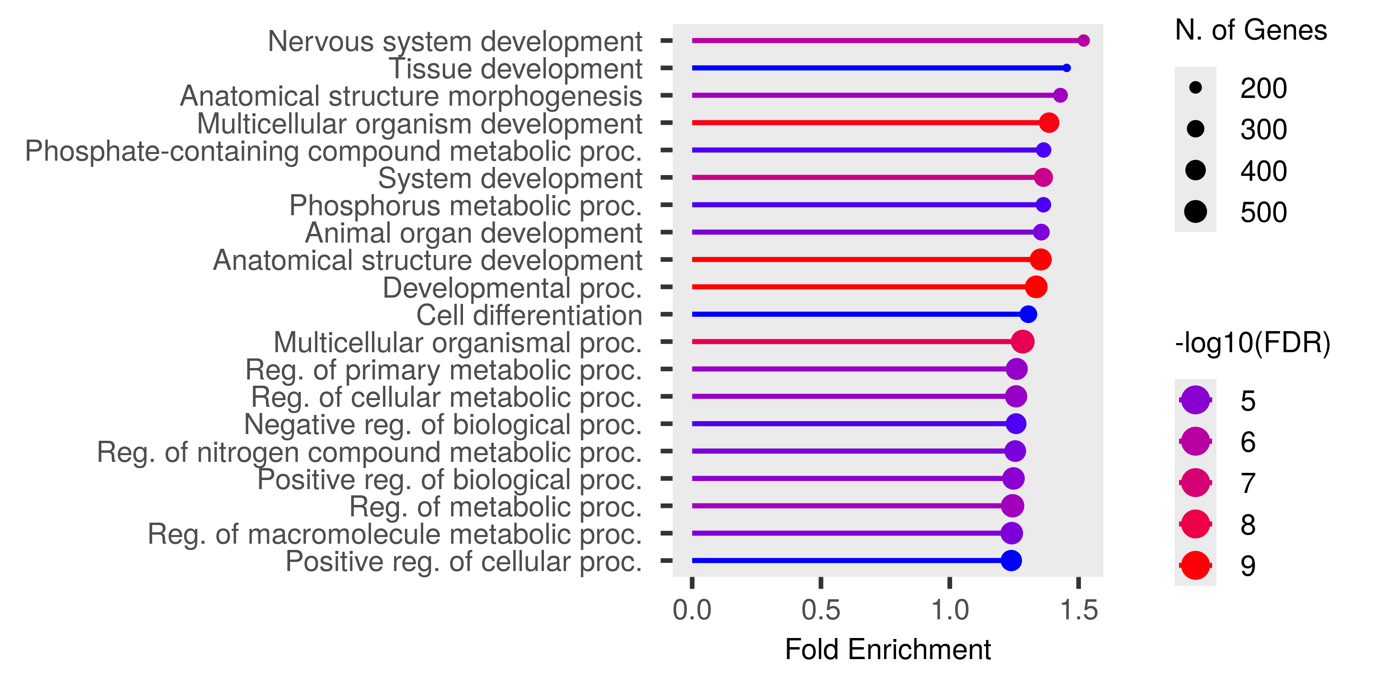
**

**Supplementary Figure 1.** Significant GO enrichment terms for the Afrikaner population

**Supplementary Figure 2.** Significant GO enrichment terms for the Bonsmara population

## Supplementary Tables

**Supplementary Table 1.** Candidate genes relating to adaptation obtained from the FST analysis between the Afrikaner and Bonsmara populations and filtered by biological relevance

| **BTA** | **Gene start ^a^** | **Gene name** | **Role** | **References** |
| --- | --- | --- | --- | --- |
| 1 | 50258772 | *CBLB* | Inflammatory response; hoof and leg disorders | Han *et al.,* 2020; Kosińska-Selbi *et al.*, 2020 |
| 5 | 103631360 | *CD4* | Upregulated in bovine virus infected mammary tissues | Çomakli & Özdemir, 2019 |
| 17 | 35062902 | *IL21* | Host-liver fluke interaction; inflammatory bowel disease | May *et al.,* 2025; Walsh *et al.,* 2009; Zwane *et al.,* 2021 |
| 5 | 103493143 | *PHB2* | Parkin- mediated mitophagy | De Falco *et al.,* 2020 |
| 5 | 103502500 | *PTPN6* | Resistance to vector-borne diseases; trypanotolerance | Kambal *et al.,* 2023; Tijjani, 2019 |
| 7 | 51762895 | *CD14* | Innate immunity and SCS | Gupta *et al.*, 2018; Xue *et al.,* 2018 |
| 9 | 59679876 | *MAP3K7* | Immune response | Boschiero *et al.,* 2025 |
| 7 | 40663606 | *NLRP3* | Immune regulation; inflammasome | Bauernfeind & Hornung, 2013; Harte *et al.,* 2017 |
| 7 | 50736654 | *STING1* | Thermogenesis; immune response; resistance to vector-borne diseases | Kim *et al.*, 2020; Huang *et al.,* 2023; Terefe *et al.,* 2023 |
| 8 | 104084879 | *TNFSF8* | Immune response | Wang *et al.,* 2020 |
| 11 | 92474734 | *DAB2IP* | QTLs associated with stillbirth | Sanchez *et al.*, 2024 |
| 10 | 34100135 | *RASGRP1* | Mastitis resistance/susceptibility | Essa *et al.*, 2023 |
| 7 | 50680729 | *DNAJC18* | Heat tolerance | Bahbahani *et al.*, 2017 |
| 7 | 50593615 | *MATR3* | Local pathogen adaptation | Kim *et al.*, 2020 |

^a^ Gene positions measured in base pairs (bp); BTA – *Bos taurus* autosome

**References**

Bahbahani, H., Tijjani, A., Mukasa, C., Wragg, D., Almathen, F., Nash, O., et al. (2017). Signatures of selection for environmental adaptation and zebu x taurine hybrid ztness in East African shorthorn Zebu. Front. Genet. 8, 68. doi:10.3389/fgene.2017.00068

Bauernfeind, F., and Hornung, V. (2013). Of inpammasome and pathogens – sensing of microbes by the inpammasome. EMBO Mol. Med. 5, 814–826. doi:10.1002/emmm. 201201771

Boschiero, C., Beshah, E., Zhu, X., Tuo, W., and Liu, G. E. (2025). Prozling genome- wide methylation patterns in cattle infected with ostertagia osstertagi. Int. J. Mol. Sci. 26, 89. doi:10.3390/ijms26010089

Çomakli, S., and Özdemir, S. (2019). Comparative evaluation of the immune responses in cattle mammary tissues naturally infected with bovine parainpuenza virus type 3 and bovine Alphaherpesvirus-1. Pathogens 8, 26. doi:10.3380/ pathogens8010026

De Falco, F., Gentile, I., Cerino, P., Cutarelli, A., Catoi, C., and Roperto, S. (2020). Prohibitin 2 is involved in Parkin-mediated mitophagy in urothelial cells of cattle infected with bovine papillomavirus. Pathogens 9, 621. doi:10.3390/pathogens9080621

Essa, B., Al-Sharif, M., Abdo, M., Fericean, L., and Ateya, A. (2023). New insights on nucleotide sequence variants and mRNA levels of candidate genes assessing resistance/ susceptibility to mastitis in Holstein and Montbéliarde dairy cows. Vet. Sci. 10, 35. doi:10.3390/vetsci10010035

Gupta, J. P., Bhushan, B., Asaf, V. N. M., Kumar, A., Ranjan, S., Panigrahi, M., et al. (2018). Association and expression analysis of single nucleotide polymorphisms of CD14 gene with somatic cell score in crossbred cattle. Gene Rep. 12, 255–260. doi:10. 1016/j.genrep.2018.07.011

Han, S., Li, X., Liu, J., Zou, Z., Luo, L., Wu, R., et al. (2020). Bta-miR-223 targeting CBLB contributes to resistance to Staphylococcus aureus mastitis through the PI3K/ AKT/NF-kB pathway. Front. Vet. Sci. 7, 00529. doi:10.3389/fvets.2020.00529

Harte, C., Gorman, A. L., McCluskey, S., Carty, M., Bowie, A. G., Scott, C. J., et al. (2017). Alum activated the bovine NLRP3 inpammasome. Front. Immunol. 8, 01494. doi:10.3389/zmmu.2017.01494

Huang, N., Zhao, L., Wang, J., Jiang, Q., Ju, Z., Wang, X., et al. (2023). Signatures of selection in indigenous Chinese cattle genomes reveal adaptive genes and genetic variations to cold climate. J. Anim. Sci. 101, skad006. doi:10.1093/jas/skad006

Kambal, S., Tijjani, A., Ibrahim, S. A. E., Ahmed, M.-K. A., Mwacharo, J. M., and Hanotte, O. (2023). Candidate signatures of positive selection for environmental adaptation in indigenous African cattle: a review. Anim. Genet. 54, 689–709. doi:10. 1111/age.13353

Kim, K., Kwon, T., Dessie, T., Yoo, D. A., Mwai, O. A., Jang, J., et al. (2020). The mosaic genome of indigenous African cattle as a unique genetic resource for African pastoralism. Nat. Genet. 52, 1099–1110. doi:10.1038/s41588-020-0694-2

Kosiqska-Selbi, B., Suchocki, T., Egger-Danner, C., Schwarzenbacher, H., Fraszczak, M., and Szyda, J. (2020). Exploring the potential genetic heterogeneity in the incidence of hoof disorders in Austrian Fleckvieh and Braunvieh cattle. Front. Genet. 11, 577116. doi:10.3389/fgene.2020.577116

May, K., Hecker, A. S., Strube, C., Yin, T., and König, S. (2025). Genetic parameters and single-step genome-wide association analysis for trematode (Fasciola hepatica and Calicophoron/Paramphistotum spp.) infections in German dairy cows. Infect. Genet. Evol. 128, 105712. doi:10.1016/j.meegid.2025.105712

Sanchez, L., Campos-Chillon, F., Sargolzaei, M., Peterson, D. G., Sprayberry, K. A., McArthur, G., et al. (2024). Molecular mechanisms associated with the development of the metritis complex in dairy cattle, Genes (Basel). 15: 439. doi:10.3390/genes15040439

Terefe, E., Belay, G., Tijjani, A., Han, J., and Hanotte, O. (2023). Whole genome resequencing reveals genetic diversity and selection signatures of Ethiopian indigenous cattle adapted to local environments. Diversity 15, 540. doi:10.3390/d15040540

Tijjani, A. (2019). Genome diversity and adaptation of African Taurine and Zebu cattle. University of Nottingham, Nottingham. PhD thesis.

Walsh, K. P., Brady, M. T., Finlay, C. M., Boon, L., and Mills, K. H. G. (2009). Infection with a helminth parasite attenuates autoimmunity through TGF-beta-mediated suppression of Th17 and Th1 responses. J. Immunol. 183, 1577–1586. doi:10.4049/ jimmunol.0803803

Wang, M., Liang, Y., Ibeagha-Awemu, E. M., Li, M., Zhang, H., Chen, Z., et al. (2020). Genome-wide DNA methylation analysis of mammary gland tissues from Chinese Holstein cows with Staphylococcus aureus induced mastitis. Front. Genet. 11, 550515. doi:10.3389/fgene.2020.550515

Xue, Y., Gao, W. N., Chen, F., Ma, B. B., Zhou, F., Hu, Z. G., et al. (2018). CD14 gene polymorphisms associated with increased risk of bovine tuberculosis in Chinese Holstein cows. Vet. J. 232, 1–5. doi:10.1016/j.tvjl.2017.11.015

Zwane, A. A., Nxumalo, K. S., Makgahlela, M. L., van Marle-Köster, E., and Maiwashe, N. (2021). Gene-set enrichment analysis of selective sweeps reveals phenotypic traits in Nguni. S. Afr. J. Anim. Sci. 51, 6. doi:10.4314/sajas.v51i6.9
